# Latent 3D Volume for Joint Depth Estimation and Semantic Segmentation from a Single Image

**DOI:** 10.3390/s20205765

**Published:** 2020-10-12

**Authors:** Seiya Ito, Naoshi Kaneko, Kazuhiko Sumi

**Affiliations:** 1 Graduate School of Science and Engineering, Aoyama Gakuin University, 5-10-1 Fuchinobe, Chuo-ku, Sagamihara, Kanagawa 252-5258, Japan; 2 Department of Integrated Information Technology, Aoyama Gakuin University, 5-10-1 Fuchinobe, Chuo-ku, Sagamihara, Kanagawa 252-5258, Japan; kaneko@it.aoyama.ac.jp (N.K.); sumi@it.aoyama.ac.jp (K.S.)

**Keywords:** multi-task learning, latent 3D volume, depth estimation, semantic segmentation

## Abstract

This paper proposes a novel 3D representation, namely, a latent 3D volume, for joint depth estimation and semantic segmentation. Most previous studies encoded an input scene (typically given as a 2D image) into a set of feature vectors arranged over a 2D plane. However, considering the real world is three-dimensional, this 2D arrangement reduces one dimension and may limit the capacity of feature representation. In contrast, we examine the idea of arranging the feature vectors in 3D space rather than in a 2D plane. We refer to this 3D volumetric arrangement as a latent 3D volume. We will show that the latent 3D volume is beneficial to the tasks of depth estimation and semantic segmentation because these tasks require an understanding of the 3D structure of the scene. Our network first constructs an initial 3D volume using image features and then generates latent 3D volume by passing the initial 3D volume through several 3D convolutional layers. We apply depth regression and semantic segmentation by projecting the latent 3D volume onto a 2D plane. The evaluation results show that our method outperforms previous approaches on the NYU Depth v2 dataset.

## 1. Introduction

Semantic 3D scene reconstruction, in which a scene is geometrically and semantically analyzed, is one of the challenging and crucial problems in the field of computer vision. Understanding a surrounding environment is valuable for many applications such as remote sensing, robotics, augmented reality, and human-computer interaction. Early studies tackled this problem using a combination of 3D reconstruction and image-based recognition techniques. For example, several methods using Simultaneous Localization and Mapping (SLAM) or Structure from Motion (SfM) with 2D semantic segmentation have been proposed [1,2,3,4].

Estimating depth and semantic labels from 2D images is a crucial step for precise semantic 3D scene reconstruction. Recently, Convolutional Neural Networks (CNNs) have achieved tremendous results in tasks such as depth estimation [5,6,7,8] and semantic segmentation [9,10,11,12]. An important advantage of CNNs is the ability to learn contextual information in an image. Standard CNNs apply a set of 2D convolutions to an input image to acquire such information in a 2D image plane. In addition, the modern approaches have focused on the elaborate design of multi-scale feature extraction [5,13], pooling [10,11,14], and upsampling [6] to acquire contextual information more effectively. The majority of the existing methods have dealt with depth estimation and semantic segmentation separately (i.e., single-task learning).

**Figure 1:**
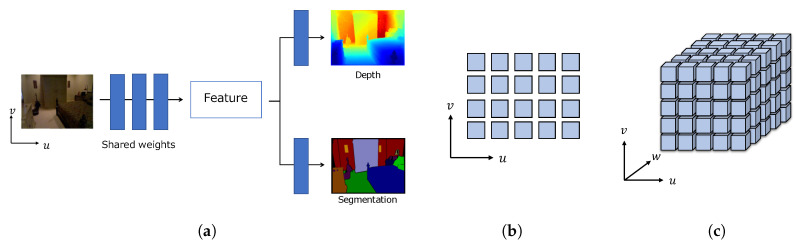
Hard parameter sharing designs for the joint depth estimation and semantic segmentation: (**a**) overview of hard parameter sharing; (**b**) previous 2D feature representation; (**c**) our proposed latent 3D volume representation. In (**b**,**c**), each rectangle or cube represents a feature vector.

In contrast, relatively few studies have addressed joint depth estimation and semantic segmentation [15,16,17,18,19,20,21]. This joint task is generally formulated as a multi-task learning problem, in which a single CNN is used to estimate both depth and semantic structure of a scene, instead of using a separate dedicated CNN for each task. The typical CNN design choice for the joint depth estimation and semantic segmentation is so-called hard parameter sharing [15,16,17,18,19,20,21]. In hard parameter sharing, a network has hidden layers shared between all tasks, while having several task-specific output layers (Figure 1a). These shared layers produce shared feature maps, which capture the feature representation useful for all of the tasks. Hence, the design of this representation is one of the key aspects of the joint problem. Previous approaches to the joint task [15,16,17,18,19,20,21] employ a set of 2D convolutions to construct the shared representation, which is given as a set of feature vectors arranged over a 2D plane (Figure 1b). However, considering the real world is three-dimensional, this 2D arrangement reduces one dimension and may limit the capacity of feature representation. Besides, a set of 2D convolutions extracts 2D spatial relationships and may be unsuitable for obtaining 3D spatial contextual information.

In this paper, we examine a novel feature representation for joint depth estimation and semantic segmentation. Considering the real world, the objects and structures are originally arranged in 3D space, and the feature maps should therefore also be represented in such a space. Our key idea is arranging feature vectors on a 3D grid (Figure 1c), where each feature vector has both geometric and semantic information. Because geometric structures and semantics are mutually related, we believe that this representation is suitable for the understanding of a 3D scene. To distinguish the proposed 3D feature representation from feature vectors arranged over a 2D plane, we refer to the proposed representation as a 3D feature volume. We construct the initial 3D feature volume using feature maps extracted by 2D CNNs. The initial volume is then regularized by 3D CNNs to acquire 3D feature volume. We refer to the acquired 3D feature volume as a latent 3D volume. The latent 3D volume is shared and used to infer depth and semantic segmentation. By training the network in an end-to-end manner, 2D CNNs are trained to output 2D feature maps to implicitly have the 3D structure in the channel dimension. As the latent 3D volume is used for depth estimation and semantic segmentation, it contains both geometric and semantic features.

There are three questions we examine in this paper:

1. How to build a latent 3D volume;
2. How to use the latent 3D volume for depth estimation and semantic segmentation; and
3. How to learn the latent 3D volume efficiently.

For the first question, we employ hard parameter sharing design and explore a network architecture to construct a latent 3D volume from images to have geometric and semantic information. We propose a projection module that projects the latent 3D volume onto a 2D plane for the second question. The proposed projection module is a two-branch structure that decomposes the latent 3D volume into geometric and semantic features. We evaluate the latent 3D volume on the NYU Depth v2 dataset [22], which has only 1449 images annotated in terms of both depth and semantic segmentation. For the third question, we also propose a training strategy using the SUNCG dataset [23], which is an indoor synthetic dataset, to deal with the small amount of annotated data. We divide the training procedure into two stages: one is a pre-training using the synthetic dataset and the other is a fine-tuning using a real-world dataset. We experimentally demonstrated that the proposed method achieves significant performance improvements over previous methods.

To the best of our knowledge, this is the first attempt to introduce a 3D representation for the joint task of depth estimation and semantic segmentation from a single image. We also propose a novel projection module which effectively passes a latent 3D volume to task-specific layers. To summarize, we present:

- A novel 3D representation that aggregates geometric and semantic information in a latent space;
- A two-branch projection module that projects a latent 3D volume onto a 2D plane and decomposes it into geometric and semantic features;
- A training strategy to effectively learn a latent 3D volume by using a large-scale synthetic dataset adapted to real domains and a small-scale real-world dataset.

The rest of this paper is organized as follows. Section 2 introduces previous studies related to this field. Section 3 describes the architecture to learn our proposed latent 3D volume. Detailed procedures to train the proposed latent 3D volume are described in Section 4. In Section 5, the experimental results on the standard NYU Depth v2 indoor scene dataset [22] are described. Further analysis of the proposed method is discussed in Section 6. Finally, Section 7 provides some concluding remarks and discusses future work.

## 2. Related Work

In this section, we review depth estimation, semantic segmentation, and their multi-task learning.

### 2.1. Depth Estimation

Early studies used hand-crafted features and probabilistic graphical models for single image depth estimation [24,25,26,27,28]. CNN-based methods have become successful in recent years.

Eigen et al. [5] proposed a robust depth estimation method using a multi-scale CNN. Owing to the learning capability of a multi-scale CNN and the availability of large-scale datasets, their latter work showed a promising performance [13]. Laina et al. [6] employed ResNet [29] as an encoder and introduced an up-projection block to effectively learn feature map upsampling. Fu et al. [8] presented a deep ordinal regression network and introduced a spacing-increasing discretization strategy that defines the continuous depth as discrete values and achieves state-of-the-art performance.

Several studies have combined a CNN with regularization based on a conditional random field (CRF) [7,30]. Liu et al. [7] tackled the problem with a deep conditional neural field, which jointly learn a CNN and a continuous CRF. Roy and Todorovic [30] showed that random forests can also be used as a regularizer.

Although these studies have discussed multi-scale feature extraction [5], feature upsampling [6], and regularization [7,30], the dimensionality of feature representations has been overlooked.

### 2.2. Semantic Segmentation

Recent semantic segmentation approaches have taken advantage of CNNs. The input modalities of semantic segmentation are categorized into RGB images [9,10,12,14,31,32,33] and RGB-D images [22,34,35,36]. Although RGB-D semantic segmentation approaches have achieved better performance than RGB semantic segmentation approaches, they assume that a precise depth map is available as an input. Thus, we focus on RGB semantic segmentation.

Recent studies [10,11,12] based on fully convolutional networks (FCNs) [9] have demonstrated significant improvements on a variety of benchmarks [22,37,38,39]. In other studies [9,31,32], the authors attempted to train deconvolution networks to overcome low-resolution estimation and leverage context information. To enhance such information, spatial pyramid pooling at several scales was presented [10], and later atrous spatial pyramid pooling applying atrous convolution with a different dilation rate was proposed [14]. Through a different approach, Lin et al. [12] presented RefineNet, which iteratively combines multi-level features to produce high-resolution estimates. Jiao et al. [33] distilled geometry-aware embeddings for semantic segmentation and achieved significant improvements over previous approaches on indoor benchmarks. Whereas our approach is similar to their approach [33] in terms of geometry-aware features, we use a 3D feature representation instead of a 2D feature representation.

### 2.3. Multi-Task Learning

Multi-task learning with the goal of learning multiple tasks simultaneously [40] is nowadays based on CNNs [13,15,16,17,18,19,20,41,42,43,44,44]. Eigen and Fergus proposed a network structure that can estimate the depth, semantic labels, and the surface orientation of a scene [13]. Kendall et al. [42] introduced a Bayesian deep learning framework combining input-dependent aleatoric uncertainty with epistemic uncertainty. Although these approaches can learn each task, training and inference are conducted separately.

There have been relatively few studies [15,16,17,18,19,20,41] on estimating the depth and semantic segmentation jointly. Wang et al. [41] tackled the problem using two separate CNNs, which obtain the regional and global potentials of a scene and feed them into a hierarchical CRF. However, a hierarchical CRF is computationally expensive. Mousavian et al. [15] estimated depth and semantic segmentation using a multi-scale shared encoder. They also showed that a fully connected CRF improves performance. Zhang et al. [21] presented a task-recursive learning framework that refines both depth and semantic segmentation recursively. Jiao et al. [20] proposed synergy network architecture that propagates semantic information to depth prediction. They also proposed an attention-driven loss to deal with long-tail data distribution. Lin et al. [17] presented a hybrid CNN that combines a common feature extraction network and a global depth estimation network for understanding the global layout of the scene. Nekrasov et al. [16] used knowledge distillation and achieved a real-time performance. Zhou et al. [18] proposed a knowledge interactiveness learning module to enclose both depth estimation and semantic segmentation information. The authors later proposed a pattern-structure diffusion framework that effectively mines and propagates the relationships within/across tasks [19].

As mentioned above, CNN-based approaches use shared feature maps to infer depth and semantic segmentation. However, these 2D representations are limited to obtain the 2D contextual information in an image due to 2D CNNs. In contrast to existing approaches [15,16,17], we propose a 3D feature representation, namely, a latent 3D volume. This representation enhances the 3D contextual information by introducing 3D CNNs. In this study, we explore the design of hard parameter sharing, whereas recent approaches [18,19,20,21] have focused on designing output layers using mutual information between tasks.

## 3. Latent 3D Volume

This section describes the details of the latent 3D volume for joint learning of depth estimation and semantic segmentation.

### 3.1. Overview

**Figure 2:**
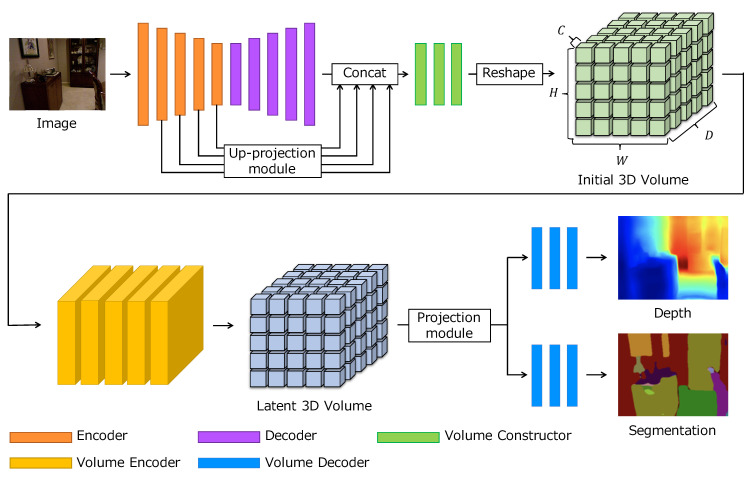
Overall architecture of our network.

Our network architecture is based on the hard parameter sharing design that combines a shared backbone and several task-specific output layers for each task. The overall architecture is illustrated in Figure 2. The proposed architecture consists of five modules: an encoder, a decoder, a volume constructor, a volume encoder, and a volume decoder. The first step is to extract deep image features using the encoder and decoder. In the proposed network, any image feature extractor can be used. In this study, we use a standard encoder-decoder network consisting of 2D convolutions [6]. The multi-scale features from the encoder are upsampled and concatenated with the output of the decoder. The volume constructor takes these concatenated features as inputs and outputs a 3D volume. To extract 3D contextual information, we pass the 3D volume through a volume encoder with several 3D convolutional layers and obtain a latent 3D volume. Finally, the latent 3D volume is fed into the volume decoder, which consists of a projection module and 2D convolutions, and both depth and semantic segmentation are inferred. The proposed network is trained to estimate depth and semantic segmentation from a single image in an end-to-end manner. As the shared backbone outputs the latent 3D volume as shared features, the latent 3D volume is learned to have both geometric and semantic information.

### 3.2. Image Features

Taking a single RGB image as an input, the encoder first extracts the features on four different scales. The final feature from the encoder is then fed into the decoder which uses the up-projection blocks consisting of unpooling and several 2D convolutions [6]. Laina et al. [6] reported that an up-projection block achieved a better performance than other upsampling methods such as deconvolution and up-convolution. The decoder first applies a 2D convolution to the encoded feature map to reduce the channel by half and gradually upsamples the obtained feature map to half the size of the input image while reducing the number of feature channels by half. The decoded feature is finally upsampled to the same size as the input image using bilinear interpolation.

In addition, the multi-scale features from the encoder are upsampled to half the size of the input image and converted to the 16-channel feature representations using the up-projection block [6]. These features are upsampled to the same size as the input image using bilinear interpolation and concatenated with the decoded features.

### 3.3. Latent Volume Generation

Taking image features as inputs, the purpose of the volume constructor is to generate a latent 3D volume of size W×H×D×C, where *W* and *H* are the width and height of the input image, *D* is the number of predefined depth samples, and *C* is the number of predefined channels. We first feed the concatenated features into a volume constructor consisting of three 2D convolutional layers with 5×5 filters. Each layer of the volume constructor yields feature maps with the same channels as the concatenated feature map except for the final layer whose output channel is of size D×C. We then generate the initial volume $V_{0}$ by reshaping the feature maps into a volume of size W×H×D×C.

The volume encoder is trained to output the latent 3D volume from the initial volume $V_{0}$. There are five 3D convolutional layers with residual blocks, which have 3×3×3 filters. Each layer yields 32-channel feature maps except for the final layer whose output dimension is *C*. We use C=32 during the experiments.

### 3.4. Depth Regression and Classification

The volume decoder infers depth and semantic segmentation. We first project the latent 3D volume *V* onto a 2D space using a projection module. Through preliminary experiments, we found that a straightforward projection module combining a 3D convolution and a squeezing of the channel dimensions is ineffective. Therefore, we explored the appropriate design of the projection module for joint depth estimation and semantic segmentation. Inspired by recent stereo matching, we use the soft argmax operation [45] as a projection module. In addition, we consider relationships between geometric structures and semantics. Thus, we design the projection module to have two branches. To preserve geometric cues, one branch produces the depth maps by conducting a 1×1×1 3D convolution and soft argmax operations. The other branch projects the latent 3D volume onto a 2D space and outputs a 2D feature map of size W×H×C. The purpose of this branch is to maintain semantic cues.

The first branch produces a depth map from the latent 3D volume *V*. We first apply a 1×1×1 3D convolution to reduce the channel dimension and obtain a 3D volume $V^{\prime }$ of size W×H×D×1. According to Im et al. [46], the discrete depth in the inverse-depth domain is more effective than the depth domain. We uniformly sample the discrete depth in the inverse-depth domain as follows:

(1) $$\begin{array}{c} d_{l}=\frac{D\times d_{min}}{l} \end{array}$$

where $d_{min}$ is the minimum scene depth and l∈{1,…,D} is the index of the depth dimension of the volume. The soft argmax operation [45] regresses a continuous depth value in the following manner:

(2) $$\begin{array}{ccc} f & = & \sum_{l=1}^{D}l\times \sigma \left(V_{l}^{\prime }\right) \end{array}$$

(3) $$\begin{array}{ccc} d & = & \frac{D\times d_{min}}{f} \end{array}$$

where σ is the softmax operation and $V_{l}^{\prime }$ is the *l*-th slice of the volume $V^{\prime }$ along with the depth dimension. This process yields the depth map with the same resolution as the input image.

The second branch generates a 2D feature map. The operation is almost the same as in the first branch, but the input is different. We slice the volume *V* along the channel dimension and then apply the soft argmax operation. All outputs of each slice are then concatenated. The resulting feature size is W×H×C.

Finally, we concatenate the outputs of each branch and use them to infer depth and semantic segmentation. We use the same architecture, which is composed of three 2D convolutional layers with 5×5 filters. The difference between depth estimation and semantic segmentation is the output dimension of the final layer, i.e., the depth estimation has a dimension of 1 and the semantic segmentation is the number of classes.

### 3.5. Loss Functions

Here, we define the loss function to train our network.

#### 3.5.1. Depth Estimation

Following the previous single depth estimation studies [5,6], we employ the sum of differences between the inferred depth D and its ground truth $\hat{D}$:

(4) $$\begin{array}{c} l_{depth}\left(D,\hat{D}\right)=\frac{1}{N}\sum_{i}e\left(i\right) \end{array}$$

where *N* represents the total number of valid pixels in the image, *i* represents the pixel, and $e\left(i\right)=|D\left(i\right)-\hat{D}{\left(i\right)|}_{1}$. Some studies use the l2 norm or burHu loss instead of the l1 norm. Ma et al. [47] reported that the berHu and l1 losses significantly outperform the l2 loss and that the l1 loss is slightly better than the berHu loss, and thus we employ the l1 norm in this study.

The depth loss is sensitive to displacement in the depth direction. To capture the structure of a scene, not only the depth of the target pixel but also the depth displacement of neighboring pixels is important. In addition to the depth loss, we employ a depth gradient loss [48] which penalizes the depth around the edges such as depth discontinuities:

(5) $$\begin{array}{c} l_{grad}\left(D,\hat{D}\right)=\frac{1}{N}\sum_{i}\left(\nabla_{x}e\left(i\right)+\nabla_{y}e\left(i\right)\right) \end{array}$$

where $\nabla_{x}$ and $\nabla_{y}$ are the spatial derivatives with respect to horizontal and vertical directions.

Although the gradient loss $l_{grad}$ penalizes areas with large depth changes, it often misses small structural errors. In contrast, the surface normal is useful for capturing local depth changes, such as surface irregularities and high-frequency waviness of the surface. To ensure fine-grained depth estimates, we also use normal loss calculated from a depth map. Let $n_{i}$ and ${\hat{n}}_{i}$ be the surface normals of the estimated depth and its ground truth, respectively. The surface normal is computed using $n_{i}={\left[-\nabla_{x}D\left(i\right),-\nabla_{y}D\left(i\right),1\right]}^{T}$, ${\hat{n}}_{i}={\left[-\nabla_{x}\hat{D}\left(i\right),-\nabla_{y}\hat{D}\left(i\right),1\right]}^{T}$. Following Hu et al. [48], the normal loss based on cosine similarity is defined as follows:

(6) $$\begin{array}{c} l_{normal}\left(D,\hat{D}\right)=\frac{1}{N}\sum_{i}\left(1-\frac{\langle n_{i},{\hat{n}}_{i}\rangle }{|n_{i}\left|\right|{\hat{n}}_{i}|}\right) \end{array}$$

where 〈·〉 is the inner product of vectors. The overall depth loss is as follows:

(7) $$\begin{array}{c} L_{depth}=l_{depth}+\lambda_{g}l_{grad}+\lambda_{n}l_{normal} \end{array}$$

The parameters $\lambda_{g}$ and $\lambda_{n}$ are both set to 1.0.

#### 3.5.2. Semantic Segmentation

For semantic segmentation, we use a pixelwise softmax classifier to predict labels for each pixel. Let S and $\hat{S}$ be the inferred segmentation and ground truth, respectively. We define the segmentation loss as follows:

(8) $$\begin{array}{c} L_{seg}=H\left(S,\hat{S}\right) \end{array}$$

where H indicates the cross-entropy loss.

#### 3.5.3. Overall Losses

Finally, our total loss is defined as follows:

(9) $$\begin{array}{c} L_{total}=L_{depth}+\lambda L_{seg} \end{array}$$

The weighting coefficient λ is set to 1.0.

## 4. Datasets and Training

### 4.1. NYU Depth v2 Dataset

We evaluate our network architecture on the standard NYU Depth v2 indoor scene depth estimation and semantic segmentation dataset [22], which has 1449 images with 40 semantic classes [49]. We use the official split for 464 scenes: 795 images from 249 scenes for training and 654 images from 215 scenes for testing.

### 4.2. SUNCG Dataset

The SUNCG dataset [23] is an indoor synthetic dataset which contains 45,622 different indoor scenes. Similar to the prior work [23], we randomly choose 30 K scenes as a training set and render 120 K color images, depth maps, and semantic labels using the camera intrinsics provided by the authors. Note that the SUNCG dataset has none of the paper, towel, or bag models included in the NYU dataset. In addition, there are few instances in some categories such as boxes.

### 4.3. Training Protocol

In joint learning of depth estimation and semantic segmentation, one of the main issues is that fewer samples for the joint task are available than for single tasks. To train our network, we use the SUNCG dataset [23] and the NYU Depth v2 dataset [22]. Our training procedure consists of two stages.

In the first stage, we pre-train the network using the SUNCG dataset [23]. Although these data are much easier to generate than manual annotations, there is a domain gap between real and synthetic data. Thus, we employ CycleGAN [50] for domain adaptation. We train the image translation between synthetic images and real images and convert all synthetic images into real images.

In the second stage, we fine-tune the entire network using the NYU Depth v2 dataset [22]. Previous studies [5,6,13,47,48] have shown that data augmentation improves performance. Similar to previous studies [5,13,48], we apply the following augmentations at random:

- *Rotation*. The RGB image, depth, and segmentation mask are rotated by r∈[−5,5] degrees.
- *Flip*. The RGB image, depth, and segmentation mask are horizontally flipped with 0.5 probability.
- *Color jitters*. Brightness, contrast, and saturation values of the RGB image are randomly scaled by c∈[0.6,1.4].

We follow the same procedure as that employed in previous studies [6,48]. We downsample images from their original size (640×480) to 320×240 using bilinear interpolation. The images are then cropped to their central parts, and we obtain images of size 304×228. The outputs of the network are the same size as the input images.

We use the same settings for each stage except for the number of iterations. We employ ResNet-50 [29] as an encoder. The minimum scene depth and the depth of volume *D* are set to 0.5 m and 16, respectively. We train the network using the SGD solver with mini-batches of size 8. The learning rate and momentum are set to 0.001 and 0.9, respectively. We pre-train the network for 150 K iterations and fine-tune the network for 30 K iterations. Our training usually takes 5 days on 4 NVIDIA Tesla V100 GPUs.

## 5. Experiments

### 5.1. Metrics

For depth estimation, we report evaluation metrics commonly used in previous studies [5,6,8,13,47,48]. Let $y_{i}$ and ${\hat{y}}_{i}$ be the predicted and ground truth depth value of a pixel *i*, and *N* be the total number of valid pixels. The metrics are defined as follows:

- Mean absolute relative difference (REL):
  (10) $$\begin{array}{c} \frac{1}{N}\sum_{i}\frac{|y_{i}-{\hat{y}}_{i}|}{y_{i}} \end{array}$$
- Root mean square error (RMSE):
  (11) $$\begin{array}{c} \sqrt{\frac{1}{N}\sum_{i}\left|\right|y_{i}-{\hat{y}}_{i}{\left|\right|}_{2}} \end{array}$$
- Mean $\log_{10}$ error (log10):
  (12) $$\begin{array}{c} \frac{1}{N}\sum_{i}\left|\log_{10}y_{i}-\log_{10}{\hat{y}}_{i}\right| \end{array}$$
- Thresholded accuracy (δ<thr):
  (13) $$\begin{array}{c} \frac{1}{N}\sum_{i}g\left(y_{i},{\hat{y}}_{i}\right) \end{array}$$
  where
  (14) $$\begin{array}{c} g\left(y_{i},{\hat{y}}_{i}\right)=\left\{\begin{array}{cc} 1 & \left(\delta <thr\right)\\ 0 & \left(otherwise\right) \end{array}\right.,\delta =\max\left(\frac{y_{i}}{{\hat{y}}_{i}},\;\frac{{\hat{y}}_{i}}{y_{i}}\right) \end{array}$$

The thresholded accuracy means the ratio of the maximum relative error δ below the threshold thr. In our experiments, we use thr=1.25 to align the setting with previous studies [6,8,13,15,16,17,42].

For semantic segmentation, we use mean intersection over union (mIoU) as a metric. For each class, the intersection over union is calculated as follows:

(15) $$\begin{array}{c} \mathrm{IoU}=\frac{TP}{TP+FP+FN} \end{array}$$

where *TP*, *FP*, and *FN* indicate true positive, false positive, and false negative, respectively. The IoU means the ratio of the area of overlap between the predicted segmentation and the ground truth (TP) and the area of the union of the predicted segmentation and the ground truth (TP+FP+FN). The mIoU is the average of all classes of IoU values.

We conducted our experiments using original images (640×480) on NVIDIA TITAN RTX with 24 GB of GPU memory.

### 5.2. Results on the NYU Depth v2 Dataset

Table 1 shows a quantitative comparison of the proposed method against previous methods including single and joint tasks. The proposed method achieves the best performance for most metrics when compared to joint task approaches [15,16,17,21]. Although Kendall et al. [42] demonstrated the best performance in terms of depth estimation error metrics, they did not use the same weights for depth estimation and semantic segmentation, and the segmentation performance was relatively poor compared to other approaches [15,16]. By contrast, the proposed method shows well-balanced results for both depth estimation and semantic segmentation, and the results of each task are comparable to those of single task methods. The main difference between our method and previous methods [15,16,17] is the shared feature representation. Specifically, the proposed method uses a 3D feature representation, whereas previous methods used 2D feature representations. The results show that the proposed 3D representation improves the performance of depth and semantic segmentation. Note that the proposed method is not applied in real-time while the method by Nekrasov et al. [16] is applied in real-time, which is 38.7 times faster than our approach (Table 2).

We also noticed that most depth estimation approaches [6,8,13] used the raw distribution of the NYU dataset, which has more than 120 K images with corresponding depth maps. Whereas Laina et al. [6] sampled the raw dataset, with 12 K unique images used for training, multi-task approaches [15,42] have used only the 795 images annotated with both depth and segmentation. Nekrasov et al. [16] sampled the raw dataset and used 25K real-world images for pre-training, fine-tuning the CNN using 795 images. Although we used 120 K images for pre-training, these images were all synthetic images. As we used only the 795 images for fine-tuning, the proposed volume can be learned from a large number of synthetic data and less than 1K real-world images instead of a large number of real-world images.

**Figure 3:**
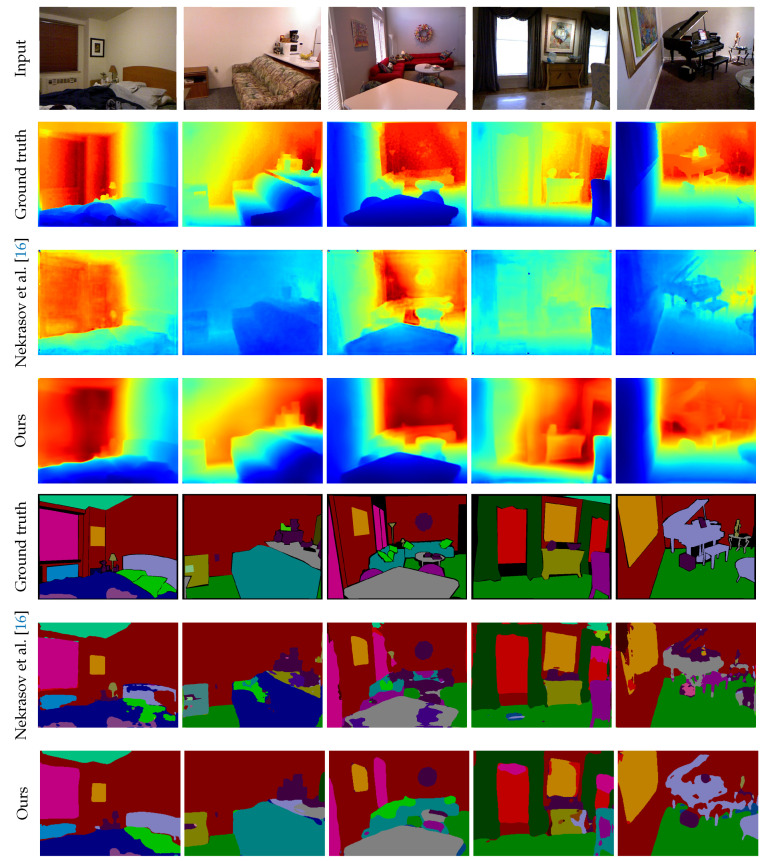
Qualitative comparison with recent joint learning approach on the NYU Depth v2 dataset. From the top to the bottom, input images, ground truth depth maps, depth results of Nekrasov et al. [16] and our proposed approach, ground truth semantic labels, and segmentation results of Nekrasov et al. [16] and our approach, respectively.

**Figure 4:**
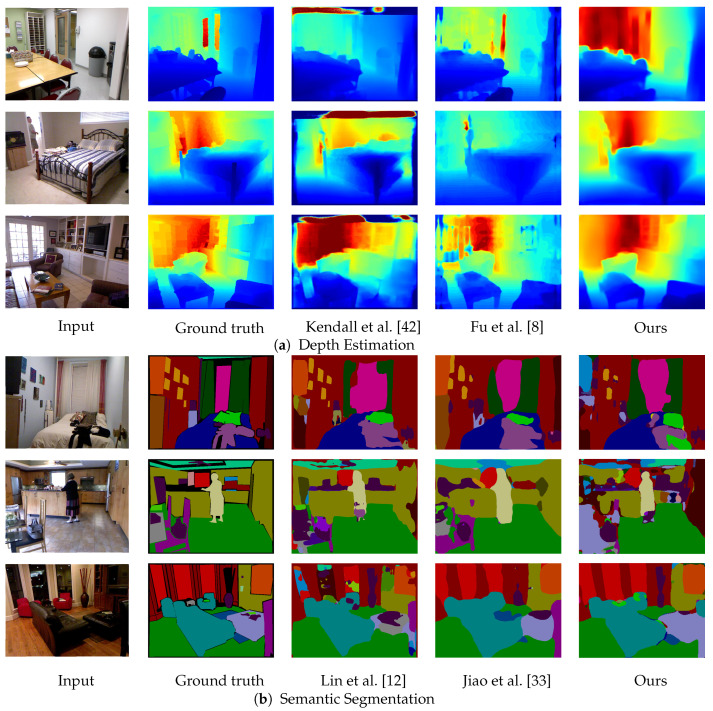
Qualitative comparison with state-of-the-art single task methods.

Figure 3 shows a qualitative comparison between our method and the method by Nekrasov et al. [16], which also employs a joint learning approach. For depth estimation, our method preserves the entire structure of the scene and correctly estimates the depth around the edges, whereas the method by Nekrasov et al. [16] often fails. For semantic segmentation, our method reduces incorrect category estimates. The latent 3D volume has more contextual information than a 2D feature map for understanding object relationships. The right two columns in Figure 3 are only the failure cases. The previous method [16] recognizes the chair in the right foreground, but our method incorrectly recognizes it as a sofa. Moreover, both methods fail to properly capture the shape of the piano. When looking at the depth results, there seems to be a lack of detailed geometry around the music stands and keys. A comparison of the results with single task methods [8,12] is shown in Figure 4. Although our approach produces smooth results for both depth and segmentation, it occasionally fails to estimate small objects or regards adjacent structures as a single region. For a more detailed analysis, a comparison with previous segmentation methods including RGB and RGB-D is shown in Table 3. Our approach was second among numerous results. As mentioned in Section 4.2, the SUNCG dataset has none of the paper, towel, or bag models, and few instances of categories such as boxes. Because our method fails with such objects, further accuracy improvement can be expected by adding these objects to the synthetic data.

### 5.3. Ablation Study

We conducted ablation studies to analyze the effects of the proposed method. The results are summarized in Table 4.

#### 5.3.1. Number of Depth Samples

**Figure 5:**
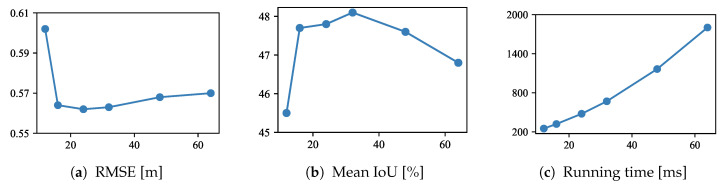
Performance with a different number of depth samples.

To study the influence of the number of depth samples *D* in the latent 3D volume, we trained models using D∈{12,16,24,32,48,64}. As shown in Figure 5a,b, the performance improves as the number of depth samples increases up to D=32. However, the proposed method derives a poor performance with D∈{48,64}. One possible reason for this is that the contextual information of 2D features extracted from the image is insufficient for constructing a 3D volume. Figure 5c shows that a greater number of depth samples requires a longer running time because the number of parameters also increases. The number of depth samples should be set depending on the architecture of the encoder and the permissible running time.

#### 5.3.2. Network Architecture

As described in Section 3.4, we analyze the effectiveness of our projection module used for inferring the depth and semantic segmentation. Table 4a shows the performance of the straightforward projection module described in Section 3.4. In addition, we validated a projection module with two different branches for depth estimation and semantic segmentation (Table 4b). This module feeds the output of each branch to a separate output layer without concatenating the output of each branch. We observed that our two-branch projection module (Table 4j) provides a better performance in the proposed network, specifically for semantic segmentation tasks.

We also investigated the design of the network architecture for the volume generation. As described in Section 3.1, the proposed network consists of several modules. Firstly, we verified the importance of the decoder. Instead of a decoder, we use a bilinear interpolation to upsample the output of the encoder. Table 4c shows that the decoder reduces the depth estimation errors and improves the mean IoU by 1.9%. Secondly, we analyze the effects of the up-projection module. Table 4d shows that the up-projection module used to extract the multi-scale features is important for constructing a latent 3D volume. Finally, we compared the model using a stacked hourglass [51] for the volume encoder. As shown in Table 4e, this slightly enhances the depth estimation results, although the segmentation results were not improved. We believe that the residual blocks provide more well-balanced results than a stacked hourglass architecture.

In addition, we analyze the difference between the depth domain and inverse-depth domain for building a 3D volume. As reported by Im et al. [46], Table 4f shows that the inverse-depth domain improves the performance of depth estimation.

#### 5.3.3. Pre-Training

To evaluate the training strategy, we compare the results with and without pre-training. As shown in Table 4g, the results show that pre-training is most effective for our network, and domain adaptation based on CycleGAN [50] further improves segmentation results (Table 4h). By contrast, the results indicate that a considerable amount of annotation data are required for greater effectiveness of a latent 3D volume representation. Compared to the knowledge distillation approach [16], a synthetic dataset provides accurate annotations. In addition, the knowledge distillation approach requires pre-training for generating pseudo semantic labels and large-scale real images. Although we train CycleGAN for domain adaptation, only small-scale real images are used for training. Thus, the proposed training strategy does not require the collection of large-scale real images.

#### 5.3.4. Single Tasks

To investigate the effects of multi-task learning, we compare the proposed method with single tasks. As shown in Table 4i, the results of depth estimation in the single-task setting are better than those of a multi-task setting. By contrast, the proposed method achieves the highest mIoU, which is 2.9% higher than that of a single-task setting. A similar relationship is observed for the method by Jiao et al. [33] because the proposed latent 3D volume likely acquires a spatial context and contributes to the semantic segmentation. Although the proposed method is accurate for multiple tasks, for a single task, it is more practical to use state-of-the-art approaches [6,12] that are faster than our approach with fewer parameters.

## 6. Discussion

As described in Section 1, there are three questions throughout this paper. In this section, we discuss how the proposed latent 3D volume is beneficial to the tasks of depth estimation and semantic segmentation.

- *Hard parameter sharing.* For the joint task of depth estimation and semantic segmentation, the typical design choice is hard parameter sharing. Although dimensionality of feature representations is an important aspect, it has been overlooked in previous studies [15,16,17,18,19,20,41]. In this study, considering the real world is three-dimensional, we proposed a latent 3D volume representation.
- *Comparison against state-of-the-art methods.* We evaluated our method on the NYU Depth v2 dataset [22]. As shown in Section 5.2, the proposed method achieves the best performance in both depth estimation and semantic segmentation compared to previous joint task methods [15,16,17]. The main difference between our method and previous methods is the representation of shared features in terms of dimensionality. While 2D representation has one feature vector for each pixel, the proposed representation assumes multiple depth planes and can maintain feature vectors for each depth. Having multiple feature vectors can enhance the 3D spatial context, which leads to dealing with ambiguity in depth and semantics. In other words, the proposed representation complements each other in geometric and semantic contexts. The experimental results show that the proposed latent 3D volume is superior to 2D feature representation for the joint task of depth estimation and semantic segmentation. One concern about the proposed method is running time and memory consumption. Although our new attempt is the use of 3D CNNs, it requires more running time and memory than 2D CNNs. Especially, the method by Nekrasov et al. [16] is much better than our method in terms of time and memory efficiency. A more compact representation of 3D features is required to reduce running time and memory consumption for practical use.
- *Network architecture.* Our network builds the initial volume using the 2D feature maps from 2D CNNs and then feeds it into 3D CNNs to obtain the latent 3D volume. While the initial volume is obtained by reshaping the 2D feature maps, our network can produce a latent 3D volume with 3D spatial contextual information. By training the network in an end-to-end manner, the 2D CNNs are trained to output the feature maps to have the 3D structure in the channel dimension. We examine how the network structure affects the learning of latent 3D volume. As shown in Section 5.3, multi-scale features from 2D CNNs are most effective to build a latent 3D volume. In contrast, the comparison of 3D CNNs shows that there is only a slight difference in performance between the residual and hourglass modules. In other words, multi-scale feature extraction in 3D CNNs has little effect.
- *Projection module.* The latent 3D volume is used for inferring depth and semantic segmentation. We design the projection module to have two branches to decompose the latent 3D volume into geometric and semantic features. The outputs of the branches are fed into task-specific layers. We show that concatenating the outputs of the two branches improves performance. In addition, the combination of latent 3D volume and projection module achieves promising results.
- *Training strategy.* We train the network in two stages using a large-scale synthetic dataset and a small-scale real-world dataset. Our experimental results show that pre-training using synthetic images is effective for learning the latent 3D volume. In addition, we use the domain adaptation to fill the gap between synthetic and real images. One of the advantages of synthetic data is accurate annotation. As real data sometimes suffers from inter-sensor distortions, calibration may be required. In contrast, synthetic data and its annotations are always aligned. We show that the performance of depth and semantic segmentation can be improved by using domain-adapted synthetic data.

## 7. Conclusions

We presented a latent 3D volume for joint depth estimation and semantic segmentation. The initial 3D volume is constructed using an encoder-decoder network and multi-scale features from the encoder. We pass the volume through several 3D convolutional layers and aggregate the geometric and semantic features in a 3D latent volume. The latent 3D volume is then used for inferring depth and semantic segmentation. We evaluated the proposed method on the NYU Depth v2 dataset and showed that our proposed latent 3D volume improves the performance of joint learning when applied to depth estimation and semantic segmentation under most metrics, and achieves comparable results to those of state-of-the-art single task methods.

We see several directions for future studies.

Firstly, more compact representations of 3D features should be explored. As the proposed 3D representation has the same height and width of the input image, it requires large memory and long running time. To reduce the cost, recent implicit functions such as [52] that encode spatial information into a low-dimensional code could be applied.

Secondly, the preparation of the training data could also be further investigated. Following previous work [23], we generated 120 K images for pre-training. Because it may be possible to pre-train with fewer images, we hope to validate the minimum number of synthetic images necessary for pre-training. In addition, we applied a domain adaptation based on CycleGAN [50] using a synthetic dataset. Instead of this approach, more recent domain adaptation [53] or knowledge distillation [54] may improve the performance.

Thirdly, the training strategy for learning latent 3D volumes could be developed. Because manual annotation is laborious, it may be desirable to train the network using different datasets for depth and semantic segmentation. Although we use real-world images with both depth and semantic segmentation annotations, depth annotations can be generated in an unsupervised manner using multiple images with overlap, and it is relatively easy to collect such real-world images. We plan to explore training strategies using different datasets.

## Figures and Tables

**Table 1:** Quantitative comparison against previous approaches on the NYU Depth v2 dataset. † denotes that two tasks use the same architecture but not the same weights. ‡ employs a single model for the joint task. The bold and underlined items represent the best and second place, respectively.

	Task		Depth Estimation				Sem. Segm.
Method	Depth	Sem. Segm.	REL ↓	RMSE ↓	log10 ↓	δ<1.25 ↑	MIoU↑
Laina et al. [6]	✓		0.127	0.573	0.055	0.811	
Fu et al. [8]	✓		0.115	0.509	0.051	**0.828**	
Lin et al. [12]		✓					46.5
Jiao et al. [33]		✓					**59.6**
† Eigen & Fergus [13]	✓	✓	0.158	0.641	-	0.769	34.1
† Kendall & Gal [42]	✓	✓	**0.110**	**0.506**	-	0.817	37.3
‡ Mousavian et al. [15]	✓	✓	0.200	0.816	-	0.568	39.2
‡ Zhang et al. [21]	✓	✓	0.144	0.501	-	0.815	46.4
‡ Lin et al. [17]	✓	✓	0.279	0.942	-	0.501	36.5
‡ Nekrasov et al. [16]	✓	✓	0.149	0.565	-	0.790	42.2
‡ Ours	✓	✓	0.139	0.564	0.059	0.819	47.7

**Table 2:** Comparison of the number of parameters and running time. Previous approaches have publicly available codes. ‘N/A’ means that a 640×480 image resolution can not be taken as an input due to the design of networks or GPU memory.

	Task		General	
Method	Depth	Sem. Segm.	Params [M]	Running Time [ms]
Laina et al. [6]	✓		63.57	21.9±1.9
Fu et al. [8]	✓			N/A
Lin et al. [12]		✓	118.10	52.6±0.1
Jiao et al. [33]		✓	103.46	N/A
Nekrasov et al. [16]	✓	✓	3.07	8.3±0.2
Ours	✓	✓	77.43	320.9±1.1

**Table 3:** Comparison of IoU with previous approaches on each category of the NYU Depth v2 dataset. * takes an RGB image as an input whereas the others take an RGB-D image. The bold and underlined items represent the best and second place, respectively.

Method	Wall	Floor	Cabinet	Bed	Chair	Sofa	Table	Door
FCN [9]	69.9	79.4	50.3	66.0	47.5	53.2	32.8	22.1
Gupta et al. [34]	68.0	81.3	44.9	65.0	47.9	47.9	29.9	20.3
Cheng et al. [35]	78.5	**87.1**	56.6	70.1	**65.2**	63.9	46.9	35.9
Park et al. [36]	**79.7**	87.0	60.9	73.4	64.6	65.4	50.7	39.9
* Jiao et al. [33]	71.4	75.2	**71.3**	**77.1**	53.3	**69.5**	**51.4**	**63.7**
* Ours	79.0	84.8	62.4	69.9	61.5	67.0	46.1	47.1
	**Window**	**Bookshelf**	**Picture**	**Counter**	**Blinds**	**Desk**	**Shelves**	**Curtain**
FCN [9]	39.0	36.1	50.5	54.2	45.8	11.9	8.6	32.5
Gupta et al. [34]	32.6	18.1	40.3	51.3	42.0	11.3	3.5	29.1
Cheng et al. [35]	47.1	48.9	54.3	66.3	51.7	20.6	13.7	49.8
Park et al. [36]	49.6	44.9	61.2	**67.1**	63.9	28.6	14.2	59.7
* Jiao et al. [33]	**68.2**	**57.3**	61.4	53.1	**77.1**	**55.2**	**52.5**	**70.4**
* Ours	53.3	52.1	**62.0**	53.1	62.6	36.7	26.4	60.0
	**Dresser**	**Pillow**	**Mirror**	**Floormat**	**Clothes**	**Ceiling**	**Books**	**Fridge**
FCN [9]	31.0	37.5	22.4	13.6	18.3	59.1	27.3	27.0
Gupta et al. [34]	34.8	34.4	16.4	28.0	4.7	60.5	6.4	14.5
Cheng et al. [35]	43.2	50.4	48.5	32.2	24.7	62.0	34.2	45.3
Park et al. [36]	49.0	49.9	54.3	39.4	26.9	69.1	35.0	58.9
* Jiao et al. [33]	**64.2**	**51.6**	**68.3**	**61.3**	**53.1**	58.1	**42.9**	**62.2**
* Ours	59.9	46.8	52.0	49.1	38.8	**71.1**	34.6	37.3
	**TV**	**Paper**	**Towel**	**Shower**	**Box**	**Board**	**Person**	**Nightstand**
FCN [9]	41.9	15.9	26.1	14.1	6.5	12.9	57.6	30.1
Gupta et al. [34]	31.0	14.3	16.3	4.2	2.1	14.2	0.2	27.2
Cheng et al. [35]	53.4	27.7	42.6	23.9	11.2	58.8	53.2	54.1
Park et al. [36]	63.8	34.1	41.6	38.5	11.6	54.0	**80.0**	45.3
* Jiao et al. [33]	**71.7**	**40.0**	**58.2**	**79.2**	**44.1**	**72.6**	55.9	**55.0**
* Ours	68.5	22.3	44.1	29.8	4.7	42.7	64.1	23.4
	**Toilet**	**Sink**	**Lamp**	**Bathtub**	**Bag**	**Ot. Struct.**	**Ot. Furn.**	**Ot. Props.**
FCN [9]	61.3	44.8	32.1	39.2	4.8	15.2	7.7	30.0
Gupta et al. [34]	55.1	37.5	34.8	38.2	0.2	7.1	6.1	23.1
Cheng et al. [35]	**80.4**	59.2	45.5	52.6	15.9	12.7	16.4	29.3
Park et al. [36]	65.7	**62.1**	**47.1**	57.3	19.1	30.7	20.6	**39.0**
* Jiao et al. [33]	72.5	50.8	33.6	**72.3**	**46.3**	**50.6**	**54.1**	37.8
* Ours	67.7	47.8	35.3	35.1	6.7	33.4	30.2	38.5

**Table 4:** Ablation experiments. The bold items represent the best place.

Method	REL	RMSE	MIoU
(a) Straightforward projection module	0.143	**0.548**	43.3
(b) Two different branches	0.130	0.563	45.7
(c) w/o Decoder	0.151	0.581	45.8
(d) w/o Up-projection module	0.154	0.583	43.1
(e) Stacked hourglass	0.132	0.567	47.2
(f) Depth domain	0.146	0.573	47.2
(g) w/o Pre-training	0.151	0.583	41.8
(h) Pre-training w/o Domain Adaptation	**0.125**	0.561	43.5
(i) Single tasks	0.127	0.559	44.8
(j) Ours	0.139	0.564	**47.7**

## References

[1] Hane C., Zach C., Cohen A., Angst R., Pollefeys M. Joint 3D Scene Reconstruction and Class Segmentation. Proceedings of the IEEE Conference on Computer Vision and Pattern Recognition (CVPR).

[2] Sengupta S., Greveson E., Shahrokni A., Torr P.H.S. Urban 3D semantic modelling using stereo vision. Proceedings of the IEEE International Conference on Robotics and Automation (ICRA).

[3] Kundu A., Li Y., Dellaert F., Li F., Rehg J.M. Joint Semantic Segmentation and 3D Reconstruction from Monocular Video. Proceedings of the European Conference on Computer Vision (ECCV).

[4] Hane C., Zach C., Cohen A., Pollefeys M. (2017). Dense Semantic 3D Reconstruction. IEEE Trans. Pattern Anal. Mach. Intell. (TPAMI).

[5] Eigen D., Puhrsch C., Fergus R. Depth Map Prediction from a Single Image using a Multi-Scale Deep Network. Proceedings of the Advances in Neural Information Processing Systems (NIPS).

[6] Laina I., Rupprecht C., Belagiannis V., Tombari F., Navab N. Deeper Depth Prediction with Fully Convolutional Residual Networks. Proceedings of the International Conference on 3D Vision (3DV).

[7] Liu F., Shen C., Lin G., Reid I. (2016). Learning Depth from Single Monocular Images Using Deep Convolutional Neural Fields. IEEE Trans. Pattern Anal. Mach. Intell..

[8] Fu H., Gong M., Wang C., Batmanghelich K., Tao D. Deep Ordinal Regression Network for Monocular Depth Estimation. Proceedings of the IEEE Conference on Computer Vision and Pattern Recognition (CVPR).

[9] Shelhamer E., Long J., Darrell T. (2017). Fully Convolutional Networks for Semantic Segmentation. IEEE Trans. Pattern Anal. Mach. Intell. (TPAMI).

[10] Zhao H., Shi J., Qi X., Wang X., Jia J. Pyramid Scene Parsing Network. Proceedings of the IEEE Conference on Computer Vision and Pattern Recognition (CVPR).

[11] Chen L., Zhu Y., Papandreou G., Schroff F., Adam H. Encoder-Decoder with Atrous Separable Convolution for Semantic Image Segmentation. Proceedings of the European Conference on Computer Vision (ECCV).

[12] Lin G., Milan A., Shen C., Reid I.D. RefineNet: Multi-path Refinement Networks for High-Resolution Semantic Segmentation. Proceedings of the IEEE Conference on Computer Vision and Pattern Recognition (CVPR).

[13] Eigen D., Fergus R. Predicting Depth, Surface Normals and Semantic Labels with a Common Multi-Scale Convolutional Architecture. Proceedings of the IEEE International Conference on Computer Vision (ICCV).

[14] Chen L., Papandreou G., Kokkinos I., Murphy K., Yuille A.L. (2018). DeepLab: Semantic Image Segmentation with Deep Convolutional Nets, Atrous Convolution, and Fully Connected CRFs. IEEE Trans. Pattern Anal. Mach. Intell..

[15] Mousavian A., Pirsiavash H., Kosecka J. Joint Semantic Segmentation and Depth Estimation with Deep Convolutional Networks. Proceedings of the International Conference on 3D Vision (3DV).

[16] Nekrasov V., Dharmasiri T., Spek A., Drummond T., Shen C., Reid I.D. Real-Time Joint Semantic Segmentation and Depth Estimation Using Asymmetric Annotations. Proceedings of the IEEE International Conference on Robotics and Automation (ICRA).

[17] Lin X., Sánchez-Escobedo D., Casas J.R., Pardàs M. (2019). Depth Estimation and Semantic Segmentation from a Single RGB Image Using a Hybrid Convolutional Neural Network. Sensors.

[18] Zhou L., Xu C., Cui Z., Yang J. KIL: Knowledge Interactiveness Learning for Joint Depth Estimation and Semantic Segmentation. Proceedings of the Asian Conference on Pattern Recognition (ACPR).

[19] Zhou L., Cui Z., Xu C., Zhang Z., Wang C., Zhang T., Yang J. Pattern-Structure Diffusion for Multi-Task Learning. Proceedings of the IEEE/CVF Conference on Computer Vision and Pattern Recognition (CVPR).

[20] Jiao J., Cao Y., Song Y., Lau R.W.H. Look Deeper into Depth: Monocular Depth Estimation with Semantic Booster and Attention-Driven Loss. Proceedings of the European Conference on Computer Vision (ECCV).

[21] Zhang Z., Cui Z., Xu C., Jie Z., Li X., Yang J. Joint Task-Recursive Learning for Semantic Segmentation and Depth Estimation. Proceedings of the European Conference on Computer Vision (ECCV).

[22] Silberman N., Hoiem D., Kohli P., Fergus R. Indoor Segmentation and Support Inference from RGBD Images. Proceedings of the European Conference on Computer Vision (ECCV).

[23] Song S., Yu F., Zeng A., Chang A.X., Savva M., Funkhouser T.A. Semantic Scene Completion from a Single Depth Image. Proceedings of the IEEE Conference on Computer Vision and Pattern Recognition (CVPR).

[24] Saxena A., Chung S.H., Ng A.Y. Learning Depth from Single Monocular Images. Proceedings of the Advances in Neural Information Processing Systems (NIPS).

[25] Saxena A., Sun M., Ng A.Y. (2009). Make3D: Learning 3D Scene Structure from a Single Still Image. IEEE Trans. Pattern Anal. Mach. Intell..

[26] Liu B., Gould S., Koller D. Single image depth estimation from predicted semantic labels. Proceedings of the IEEE Conference on Computer Vision and Pattern Recognition (CVPR).

[27] Hoiem D., Efros A.A., Hebert M. (2005). Automatic Photo Pop-up. ACM Trans. Graph..

[28] Li B., Shen C., Dai Y., van den Hengel A., He M. Depth and surface normal estimation from monocular images using regression on deep features and hierarchical CRFs. Proceedings of the IEEE Conference on Computer Vision and Pattern Recognition (CVPR).

[29] He K., Zhang X., Ren S., Sun J. Deep Residual Learning for Image Recognition. Proceedings of the IEEE Conference on Computer Vision and Pattern Recognition (CVPR).

[30] Roy A., Todorovic S. Monocular Depth Estimation Using Neural Regression Forest. Proceedings of the IEEE Conference on Computer Vision and Pattern Recognition (CVPR).

[31] Badrinarayanan V., Kendall A., Cipolla R. (2017). SegNet: A Deep Convolutional Encoder-Decoder Architecture for Image Segmentation. IEEE Trans. Pattern Anal. Mach. Intell..

[32] Ronneberger O., Fischer P., Brox T. U-Net: Convolutional Networks for Biomedical Image Segmentation. Proceedings of the Medical Image Computing and Computer-Assisted Intervention (MICCAI).

[33] Jiao J., Wei Y., Jie Z., Shi H., Lau R.W.H., Huang T.S. Geometry-Aware Distillation for Indoor Semantic Segmentation. Proceedings of the IEEE Conference on Computer Vision and Pattern Recognition (CVPR).

[34] Gupta S., Girshick R.B., Arbeláez P.A., Malik J. Learning Rich Features from RGB-D Images for Object Detection and Segmentation. Proceedings of the European Conference on Computer Vision (ECCV).

[35] Cheng Y., Cai R., Li Z., Zhao X., Huang K. Locality-Sensitive Deconvolution Networks with Gated Fusion for RGB-D Indoor Semantic Segmentation. Proceedings of the IEEE Conference on Computer Vision and Pattern Recognition (CVPR).

[36] Park S., Lee S., Hong K. RDFNet: RGB-D Multi-level Residual Feature Fusion for Indoor Semantic Segmentation. Proceedings of the IEEE International Conference on Computer Vision (ICCV).

[37] Everingham M., Eslami S.M.A., Gool L.V., Williams C.K.I., Winn J.M., Zisserman A. (2015). The Pascal Visual Object Classes Challenge: A Retrospective. Int. J. Comput. Vis..

[38] Cordts M., Omran M., Ramos S., Rehfeld T., Enzweiler M., Benenson R., Franke U., Roth S., Schiele B. The Cityscapes Dataset for Semantic Urban Scene Understanding. Proceedings of the IEEE Conference on Computer Vision and Pattern Recognition (CVPR).

[39] Zhou B., Zhao H., Puig X., Fidler S., Barriuso A., Torralba A. Scene Parsing through ADE20K Dataset. Proceedings of the IEEE Conference on Computer Vision and Pattern Recognition (CVPR).

[40] Caruana R. Multitask Learning: A Knowledge-Based Source of Inductive Bias. Proceedings of the 35th International Conference Machine Learning (ICML).

[41] Wang P., Shen X., Lin Z., Cohen S., Price B., Yuille A.L. Towards unified depth and semantic prediction from a single image. Proceedings of the IEEE Conference on Computer Vision and Pattern Recognition (CVPR).

[42] Kendall A., Gal Y. What Uncertainties Do We Need in Bayesian Deep Learning for Computer Vision?. Proceedings of the Advances in Neural Information Processing Systems (NIPS).

[43] Yin Z., Shi J. GeoNet: Unsupervised Learning of Dense Depth, Optical Flow and Camera Pose. Proceedings of the IEEE Conference on Computer Vision and Pattern Recognition (CVPR), Salt Lake City.

[44] Chen P., Liu A.H., Liu Y., Wang Y.F. Towards Scene Understanding: Unsupervised Monocular Depth Estimation With Semantic-Aware Representation. Proceedings of the IEEE Conference on Computer Vision and Pattern Recognition (CVPR).

[45] Kendall A., Martirosyan H., Dasgupta S., Henry P. End-to-End Learning of Geometry and Context for Deep Stereo Regression. Proceedings of the IEEE International Conference on Computer Vision (ICCV).

[46] Im S., Jeon H., Lin S., Kweon I.S. DPSNet: End-to-end Deep Plane Sweep Stereo. Proceedings of the 7th International Conference on Learning Representations (ICLR).

[47] Ma F., Karaman S. Sparse-to-Dense: Depth Prediction from Sparse Depth Samples and a Single Image. Proceedings of the IEEE International Conference on Robotics and Automation (ICRA).

[48] Hu J., Ozay M., Zhang Y., Okatani T. Revisiting Single Image Depth Estimation: Toward Higher Resolution Maps With Accurate Object Boundaries. Proceedings of the IEEE Winter Conference on Applications of Computer Vision (WACV).

[49] Gupta S., Arbelaez P., Malik J. Perceptual Organization and Recognition of Indoor Scenes from RGB-D Images. Proceedings of the IEEE Conference on Computer Vision and Pattern Recognition (CVPR).

[50] Zhu J., Park T., Isola P., Efros A.A. Unpaired Image-to-Image Translation Using Cycle-Consistent Adversarial Networks. Proceedings of the IEEE International Conference on Computer Vision (ICCV).

[51] Chang J.R., Chen Y.S. Pyramid Stereo Matching Network. Proceedings of the IEEE Conference on Computer Vision and Pattern Recognition (CVPR).

[52] Park J.J., Florence P., Straub J., Newcombe R.A., Lovegrove S. DeepSDF: Learning Continuous Signed Distance Functions for Shape Representation. Proceedings of the IEEE Conference on Computer Vision and Pattern Recognition (CVPR).

[53] Hoffman J., Tzeng E., Park T., Zhu J., Isola P., Saenko K., Efros A.A., Darrell T. CyCADA: Cycle-Consistent Adversarial Domain Adaptation. Proceedings of the International Conference on Machine Learning (ICML).

[54] Liu Y., Chen K., Liu C., Qin Z., Luo Z., Wang J. Structured Knowledge Distillation for Semantic Segmentation. Proceedings of the IEEE Conference on Computer Vision and Pattern Recognition (CVPR).

